# Fungal enzyme degradation of lignin-PLA composites: Insights from experiments and molecular docking simulations

**DOI:** 10.1016/j.heliyon.2023.e23838

**Published:** 2023-12-17

**Authors:** Esakkiammal Sudha Esakkimuthu, Veerapandian Ponnuchamy, Marica Mikuljan, Matthew Schwarzkopf, David DeVallance

**Affiliations:** a InnoRenew CoE, Livade 6a, 6310, Izola, Slovenia; b University of Primorska, Andrej Marušič Institute, Muzejski trg 2, 6000, Koper, Slovenia; c University of Primorska, Faculty of Mathematics, Natural Sciences and Information Technologies, Glagoljaška 8, 6000, Koper, Slovenia

**Keywords:** Biodegradation, Lignin, Polylactic acid, *Trametes versicolor*, Molecular docking

## Abstract

Fungal enzymes are effective in degrading various polymeric materials. In this study, we assessed the initial degradation of composites consisting of lignin-poly(lactic acid) (PLA) with both unmodified lignin (LIG) and oxypropylated lignin (oLIG) incorporated at 10 % and 40 % weight within the PLA matrix in a fungal environment. *Trametes versicolor* fungi were used, and the samples were treated only for eight weeks. Although there was no significant difference in weight loss, the degradation process impacted the chemical and thermal properties of the composites, as shown by Fourier transform infrared spectroscopy (FTIR) and Differential scanning calorimetry (DSC) analyses. After the degradation process, the carbonyl index values decreased for all composites and the hydroxyl index values increased for LIG/PLA and a reverse trend was observed for oLIG/PLA composites. The first heating scan from DSC results showed that the melting peak and the cold crystallization peak disappeared after the degradation process. Microscopic analysis revealed that LIG/PLA exhibited higher roughness than oLIG/PLA. Molecular docking simulations were carried out using guaiacylglycerol-β-guaiacyl ether (GGE) and lactic acid (LA) as model compounds for lignin and PLA, respectively, with laccase (Lac) enzyme for *Trametes versicolor*. The docking results showed that GGE had the strongest binding interaction and affinity with *Lac* than lactic acid and oxypropylated GGE. The oxypropylated GGE formed a shorter hydrogen bonding with the *Lac* enzyme than GGE and LA. The trend associated with the degradation of composites from experimental and molecular docking findings was consistent. This combined approach provided insights into the degradation process using fungi and had the potential to be applied to different polymeric composites.

## 1 Introduction

Poly(lactic acid) (PLA) is a biodegradable polyester synthesized via polycondensation and ring-opening polymerization of lactide monomer that is initially derived from the fermentation of sugar, corn, and potato feedstocks [1]. Due to its thermoplasticity, good transparency, relative toughness, and sustainability, PLA has been widely used in various medical and disposable applications, packaging, and automobiles [2]. However, the poor UV barrier properties, thermal properties, and cost limit the PLA's applications [3]. Various efforts have been proposed to enhance the properties of PLA, including incorporating plasticizers and blending PLA with different polymers. These composites exhibit improved thermal and mechanical properties, but adding such materials affects the degradation mechanism of the resulting composites. Therefore, investigating the degradability of these composites is essential.

Lignin is the second most abundant aromatic biopolymer and possesses many advanced properties and characteristics including antioxidant, antibacterial, anti-ultraviolet properties, and hydrophilicity, leading to its utilization in polymer composites. In particular, lignin is used as a filler in the PLA matrix to improve UV barrier and thermal properties and reduce total cost [[4], [5], [6], [7], [8], [9], [10]]. Lignin is composed of three basic monomer units: coniferyl, sinapyl, and *p*-coumaryl alcohols. However, the direct incorporation of a lignin polymer in the PLA matrix results in aggregation issues. Several chemical modification reactions, such as acetylation and oxypropylation have been employed to enhance the compatibility and processability of lignin with PLA. Tuning the original lignin structure enhances the final properties of PLA composites, but the degradation of these composite materials remains a major concern.

PLA can be decomposed in soil and dedicated composting environments. Still, the degradation rate in the soil is slower due to the low temperature and water content that lead to slow hydrolysis and low concentration of degradation microorganisms [11]. In contrast, the composting environment accelerates the rate of PLA degradation because the temperature (50–60 °C) is higher than the ambient environment, causing hydrolysis of PLA into corresponding monomer and oligomer molecules, which are followed by simultaneous degradation products such as CO_2_ and H_2_O [12,13]. PLA degradation is even faster in unsterile compost conditions, containing a mixture of fungi and bacteria, and the results suggest a higher synergy between fungi and bacteria due to diverse enzymes released in the medium [14].

Fungal enzymes can effectively degrade a variety of polymeric materials. This process involves breaking down the polymer chains into smaller units that the fungus can utilize as a source of nutrition. Such an approach for polymer degradation is environmentally friendly compared to traditional chemical methods. Several methods and fungal enzymes have been proposed to investigate the degradability of PLA composites, including soil burial [[15], [16], [17], [18], [19]], and fungal [14,19] methods. A garden soil test was performed on LIG/PLA composites with different weight percentages of lignin content and found that 10 % lignin in PLA exhibited higher fractures compared to 5 % lignin content in PLA. Triwulandari et al. carried out a degradation study on the effect of lignin in PLA composites using *Aspergillus niger* and found that increasing the lignin content in PLA composites enhanced the weight loss of biocomposites [20]. These studies have shown that fungi accelerate the degradation of lignin and PLA composites. A recent review mentioned that bacteria perform poorly compared to fungi when lignin is present [21]. Among different fungal species, white-rot fungal systems like *Trametes* sp.*, Heterobasidion* sp., and *Phanerochaete* sp. are potential candidates to degrade lignin more efficiently than other fungal systems, including brown-rot and soft-rot. In particular, *Trametes versicolor* fungi produce an enzyme, called laccase, that predominantly degrades phenolic compounds like lignin [22]. This particular enzyme has been applied successfully for various materials including aromatic polymers and dyes [[23], [24], [25], [26], [27], [28]] as the authors reported that the active laccase enzyme in the *Trametes versicolor* is capable of degrading these materials efficiently. This enzyme contains active sites, containing four copper ions where the substrate (e.g., polymers) binds and where the enzyme-catalyzed chemical reactions, especially oxidoreductive, occur during the degradation process. Therefore, studying these active sites in the enzymes is crucial to understanding the typical interaction between the enzymes and substrate. Molecular docking is a molecular modeling method that provides a clear understanding of enzyme interactions with polymer composites at the atomic level, characterizing the underlying mechanisms involved during the degradation process. This method has been successfully applied to study the polymer degradation pathway of polyethylene [29] and can be used to predict substrate position with the enzyme's active site, binding affinity, and binding interactions [[30], [31], [32], [33]].

Our previous work [34] investigated the incorporation of lignin and oxypropylated lignin at various weight percentages in PLA matrix to improve thermal and mechanical properties for sustainable-based packaging. The results showed that oxypropylation significantly enhanced PLA properties, making the composites suitable for sustainable packaging applications. However, it is crucial to evaluate the degradation of these composites for their sustainability, which is the focus of our current study.

In this work, we investigate the initial degradation of unmodified lignin (LIG)/PLA and oxypropylated lignin (oLIG)/PLA composites using *Trametes versicolor*, a white-rot fungus that has been used for the degradation of polymer composites. The composites were incubated for only eight weeks under a fungal medium, and weight loss was measured to discover the initial degradation of polymer composites. Differential scanning calorimetry (DSC) and Fourier transform infrared spectroscopy (FTIR) analyses were employed to investigate the composites’ physical and chemical properties before and after the degradation process, and the surface analysis was performed using a digital microscope. For understanding the initial steps of degradation, molecular docking modeling was carried out to understand the interaction of composites with enzymes at the atomic scale. The binding affinity, inhibition constant, and hydrophobic surface of laccase enzyme (*Lac*) from *Trametes versicolor* with lignin model compound guaiacylglycerol-β-guaiacyl ether (GGE) for unmodified and oxypropylated GGE (oGGE), and lactic acid were calculated to propose a degradation mechanism and support experimental findings.

## 2 Materials and methods

### 2.1 Chemicals

Kraft softwood lignin (UPM Biopiva 395) was purchased from UPM biochemicals with an average molecular weight of 6000 g/mol and less than 2 % ash content. Polylactic acid (PLA) pellets, namely Inzea F38 were purchased from the Nurel biopolymers and contained 70 % biobased content with a density of 1.23 g/cm^3^ and a melt flow index of 1.8 g/10 min and was measured with ISO 1133. NaOH (reagent grade, ≥98 % purity) and propylene carbonate (anhydrous, 99.7 % purity) were purchased from Sigma-Aldrich. Potato Dextrose Agar (PDA) purchased from Liofilchem (Teramo, Italy) was utilized as a medium culture for fungal inoculation.

### 2.2 Microorganisms and culture conditions

In this study, *Trametes versicolor* (isolated) culture was prepared [35] to determine the degradation of LIG/PLA composites. Potato Dextrose Agar (PDA) was selected as a growth medium for plating. Per manufacturer instructions, 42 g of PDA was mixed with 1 L of distilled water and sterilized for 20 min at 121 °C and then aseptically poured into plastic Petri dishes, up to a depth of 0.5 cm, resulting in approximately 25 mL of PDA in each Petri dish.

Inoculum was taken from 7-day-old cultures of *Trametes versicolor*, growing at 25 °C and relative humidity (RH) 92 %. A mycelium of cultures (6 mm × 6 mm) was placed in the center of the prepared media. Twelve petri dishes with *Trametes versicolor* were prepared. The fungi were then incubated in the dark for 7 days at 25 °C and RH 92 %, after which the composite samples were placed on top of mycelium for eight weeks.

### 2.3 The composite sample preparation

The lignin (LIG) and oxypropylated lignin (oLIG) incorporated PLA composites were considered in this study with different weight % of LIG and oLIG in PLA such as 10 %, and 40 %. A detailed synthesis procedure of oxypropylation of lignin and preparation of composite specimens and their corresponding thermal and mechanical properties can be found in our previous work [34]. In brief, the oxypropylation reaction was carried out with Kraft lignin, in which 5 g of Kraft lignin was placed in the 250 mL round bottom flask. Then, 30 g of propylene carbonate and 0.133 g of NaOH were added to the lignin and stirred using a magnetic stirrer at 170 °C for 3 h. After the reaction time, the oxypropylated lignin (oLIG) product was recovered through precipitation using acidified water (pH = 2) and filtered through a 25 mm membrane of cellulose with a 0.45 μm of pore size. The unmodified (LIG) and modified lignin (oLIG) were blended with PLA separately at different weight percentages (1 %, 5 %, 10 %, 20 %, and 40 %) and followed by injection molding. The hydroxyl group content in the LIG and oLIG was measured using ^31^P NMR. The morphology characterization using scanning electron microscopy (SEM), thermal properties using combined thermogravimetric analysis (TGA) and DSC, mechanical properties using the Zwick universal test machine, and antioxidant properties were investigated in detail [34].

### 2.4 The calculation of weight loss

To determine the rate of decay, composite samples (approximately 200 mg) were cut and sterilized using a lab dryer at 50 ± 2 °C for 48 h. Then 3 blocks were put on the plastic net and then exposed to the monoculture fungus in each Petri dish (90 mm diameter) with PDA for eight weeks. The experiment was conducted using three replicates for the incubation period. Test samples were kept at 26 °C and 92 % relative humidity (RH). After incubation, the samples were harvested, and mycelia were carefully removed from the samples’ surfaces. Samples were dried at 50 ± 2 °C, and after that, samples were weighed with a precision of 0.0001 g, and the final weight was recorded to calculate the mass loss (*M*_*i*_) according to the following equation:

$$M_{i}\left(\%\right)=\frac{M_{1}-M_{0}}{M_{0}}\times 100$$

where *Mi* is mass loss (%), *M*_*0*_ is the dry mass before decay (g); and *M*_*1*_ is the dry mass after decay (g).

### 2.5 Fourier transform infrared spectroscopy (FTIR)

FTIR spectra were recorded using a PerkinElmer spectrophotometer for the PLA, LIG/PLA, and oLIG/PLA samples before and after degradation treatment with *Trametes versicolor*. The spectra of the samples were collected in the range of 4000–400 cm^−1^ in the attenuated total reflectance mode, and a total of 64 scans with a resolution of 4 cm^−1^ were collected in transmission mode.

To quantitatively assess the oxidation of polymer composites before and after the degradation process, we calculated the carbonyl and hydroxyl indexes from FTIR absorbance spectra. The carbonyl index was determined by the ratio of the C=O peak at 1715 cm^−1^ to the C–H stretching peak at 1465 cm^−1^. Similarly, the hydroxyl index was computed as the ratio of the maximum absorbance in the range of 3600 cm^−1^ to 3100 cm^−1^ for O–H stretching to the absorbance of the C–H stretching at 1472 cm^−1^ [36]. The index value calculation was performed from three independent FTIR spectra and the standard deviation of each index value was included.

### 2.6 Differential scanning calorimetry (DSC)

DSC was performed using a DSC from TA Waters Instruments, New Castle, DE, USA, of PLA, LIG/PLA, and oLIG/PLA biocomposites before and after fungal treatments were carried out in Tzero Aluminum pans under a nitrogen atmosphere with a temperature range of −25 °C–210 °C at a heating rate of 10 °C min^−1^.

### 2.7 Microscopy analysis

Morphological and optical properties were determined using a VHX-6000 digital microscope, Keyence, Osaka, Japan. The growth of mycelium in the material and the morphology of the degraded composites were observed.

### 2.8 Ligand preparation for molecular docking

**Fig. 1:**
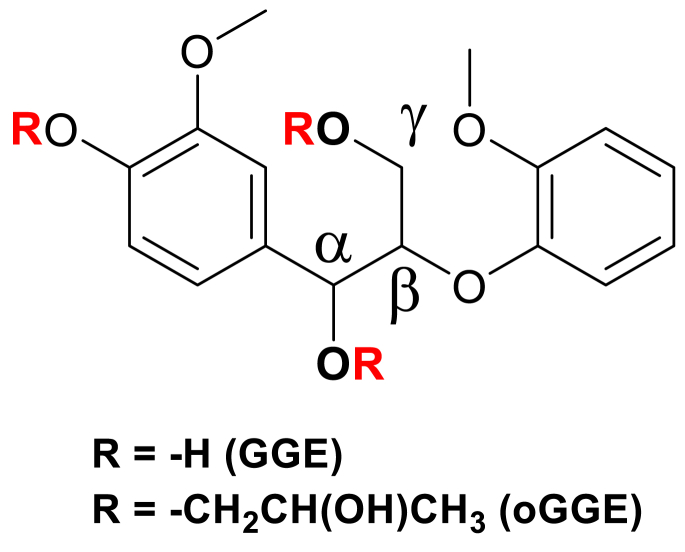
The molecular model of ligands used in this study, lignin GGE model (unmodified) and oxypropylated GGE model (oGGE).

Considering the lignin and polylactic acid's polymer structure, docking simulations with the enzyme are more demanding. Hence, we consider the typical model compound for lignin, called guaiacylglycerol-β-guaiacyl ether (GGE), which consists of the most predominant linkage, β-*O*-4 (Fig. 1). This particular linkage presents over 45 % in the lignin macromolecule compared to other linkages such as α-*O*-4, β-β, β-1 and 5-5’ [37]. The GGE model shown in Fig. 1 contains guaiacyl units with hydroxyl groups at α- and γ-positions. This GGE model has been used to represent the lignin model for quantum chemical density functional calculations (DFT) for various studies, including lignin dissolution in different solvents [[38], [39], [40]] and bond dissociation mechanisms [[41], [42], [43], [44]]. The GGE model compound was oxypropylated (oGGE) at the hydroxyl group positions to study the binding interaction with the enzyme. The lactic acid (LA) monomer was selected for docking in the case of PLA polymer. All isolated geometries were optimized in the gas phase using dispersion-corrected functional, wB97X-D with 6–311 g(d,p) basis set as depicted in reference [45]. After geometry optimization, the eigenfrequency values for all final geometries were evaluated to ensure no negative frequency in the potential energy surface. All DFT calculations were carried out using open-source the General Atomic and Molecular Electronic Structure System-US (GAMESS-US) code [46]. The optimized geometries were visualized and structured using the *.pdb file format used by the Avogadro GUI [47], which was then imported to Autodock tools [48] to prepare the ligand with *.pdbqt file format, and Gasteiger partial charges were added [49]. The tool also sets up the torsional angle between rotatable bonds, which allows the ligands to bind into the enzyme's active site as close to the realistic surroundings.

### 2.9 Enzyme preparation and molecular docking simulations

In this degradation study, an active laccase enzyme from *Trametes versicolor* fungi was selected and retrieved from the RCSB database (https://www.rcsb.org/) under the code 1KYA. The 1KYA enzyme was imported to Autodock tools, and in particular, the “*A*” chain was considered for the enzyme interaction with ligands among four chains in the structure. The water molecules and the complex ligands present in the enzyme structure were removed. The missing bonds in the amino acids were repaired with the missing bond option in the tools, and Kollmann partial charges were added. In the laccase enzyme, copper ions play a vital role in the degradation of polymers, and therefore, we include these copper ions for the rigid docking simulations. The *.pbbqt file was created for the enzyme, and blind molecular docking was performed with ligand as no active site was anticipated. The largest possible docking box size was created with a dimension of 126 × 126 × 126 Å^3^ with a grid spacing of 0.4 Å to study the binding interactions extensively. The Lamarckian genetic algorithm (LGA) was employed for 50 independent ligand runs with a maximum of 25000000 energy evaluations and 27000 evaluations [50]. The most stable binding confirmation was extracted after docking with Autodock tools by comparing the binding energies obtained from the docking simulations and converted to *.pdb file using the openbabel application and subsequently used for hydrophobic surface evaluation using UCSF Chimera [51]. The amino acids involved with ligand interaction for hydrophilic and hydrophobic coordination were visualized using LigPlot + [52]. A protein-ligand interaction profiler was used to calculate the hydrogen bond and hydrophobic distance between the amino acids involved in the interaction with ligands [53].

## 3 Results and discussion

### 3.1 Analysis of weight loss

We investigated the efficiency of *Trametes versicolor* for degrading LIG/PLA and oLIG/PLA composites by measuring the weight loss after eight weeks of fungal treatment. Fig. S1 shows the samples inoculated in the Petri dishes after the degradation process, which was covered by fungi, indicating the activity of *Trametes versicolor*. The mycelia on the samples were removed, and the samples were dried at 50 °C before weighing. We used this temperature for drying as the glass transition temperature of PLA is around 60 °C, and increasing the temperature might impact the PLA in the composites, influencing the biodegradation results. The calculated weight loss results indicated no significant difference in weight before and after the degradation process, with weight losses ranging from 3.5 % to 4.2 % for all samples, confirming that only the initial phase of the degradation took place in this study's short experimental time of 8 weeks. However, we extended our analysis of the samples using FTIR and DSC techniques to understand their impact on chemical and thermal properties after this initial degradation.

### 3.2 Chemical (FTIR) and thermal analysis (DSC) of composites

**Fig. 2:**
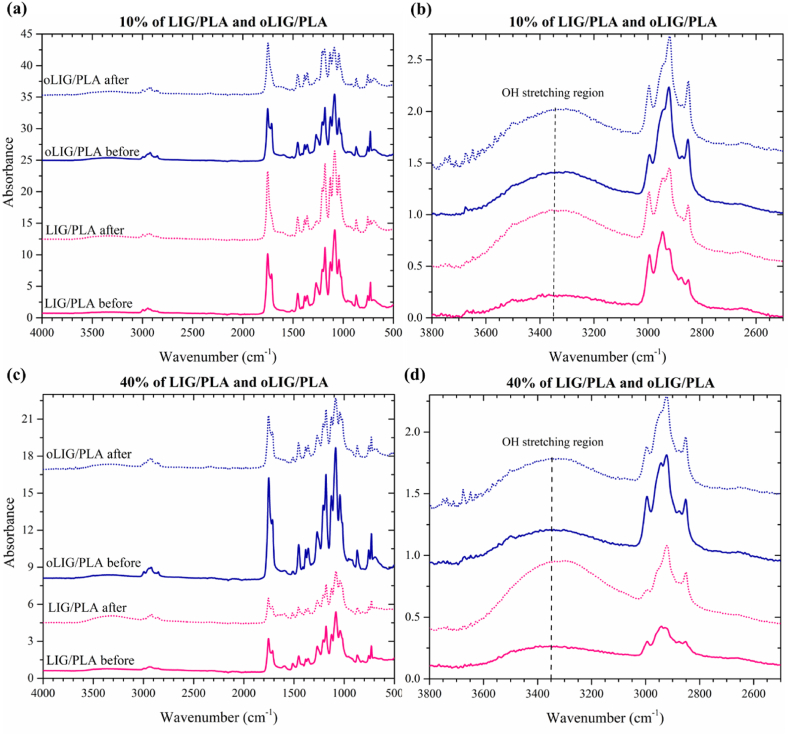
FTIR spectra of before and after the degradation process of 10 % and 40 % of LIG/PLA and oLIG/PLA composites (a) and (b).

FTIR analysis was conducted to evaluate the changes in the structure of the LIG/PLA and oLIG/PLA composites after degradation and the FTIR spectra of both 10 % and 40 % composites are presented in Fig. 2. As seen in Fig. 2b and d, the absorption band region between 3600 cm^−1^ and 3100 cm^−1^, related to the broad –OH stretching frequency is affected after the treatment. The intensity of the OH band in LIG with PLA composites significantly increased around the OH region (3350 cm^−1^) after the degradation process, indicating that lignin underwent oxidative cleavage and broke down into lignin molecules containing hydroxyl groups. The formation of hydroxyl groups in unmodified LIG/PLA composites was relatively higher than that in oLIG/PLA composites, indicating low degradation of oLIG.

**Fig. 3:**
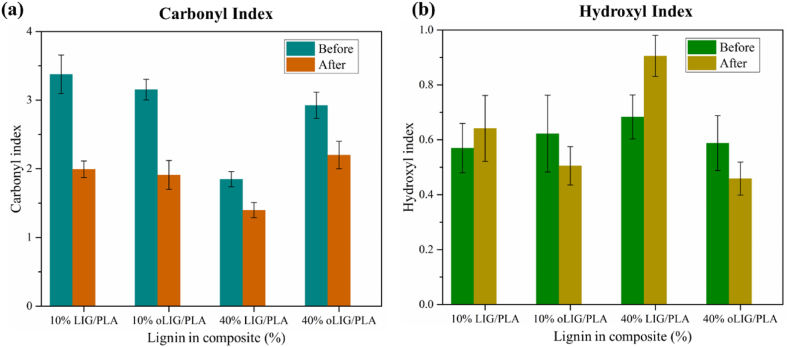
(a) The carbonyl index and (b) hydroxyl index bar plots of LIG/PLA and oLIG/PLA composites with 10 % and 40 % before and after fungal treatment.

To gain further insight into the biodegradation mechanism facilitated by fungal enzymes, we calculated the carbonyl and hydroxyl indexes using FTIR absorbance spectra. Fig. 3 illustrates the carbonyl and hydroxyl index values obtained before and after fungal treatment. Regarding the carbonyl index, a large difference was pronounced in the case of unmodified LIG/PLA composites compared to oLIG/PLA composites. The fungal enzyme, particularly laccase, actively facilitated the oxidative degradation of carbonyl group bonds, leading to a decrease in the carbonyl index values. In contrast, in the case of oLIG/PLA composites, the observed minor differences in the carbonyl index indicate that the oxypropylation reaction preserves the carbon-based structure and inhibits the oxidative degradation reaction by the laccase enzyme. Furthermore, the oxypropylation modification of lignin appears to render the composites less susceptible to fungal-induced changes in the carbonyl index properties, where the introduced oxypropyl groups might act as protective moieties, reducing the extent of degradation compared to their unmodified counterparts.

On the contrary, concerning hydroxyl index values, unmodified LIG/PLA composites, both at 10 % and 40 %, demonstrated a higher susceptibility to degradation by *Trametes versicolor* compared to their corresponding modified counterparts (oLIG/PLA). This susceptibility can be attributed to the breakdown of unchanged lignin (LIG) and polylactic acid (PLA) into smaller molecular fragments. The reduction in hydroxyl index values for oLIG/PLA composites after the degradation process suggests that the oxypropylation reaction is less effective at breaking down these composites, resulting in the formation of a lower number of hydroxyl groups. A higher concentration (40 %) of lignin (LIG) provides more substrate for fungal enzymes, intensifying the degradation effect and, consequently, influencing the higher hydroxyl-based index compared to 10 %. In the case of oLIG/PLA composites, the relative variation of obtained hydroxyl index values exhibited similar values, indicating that the concentration has no significant impact on degradation due to the low feasibility of degradation of the oxypropylated lignin.

**Fig. 4:**
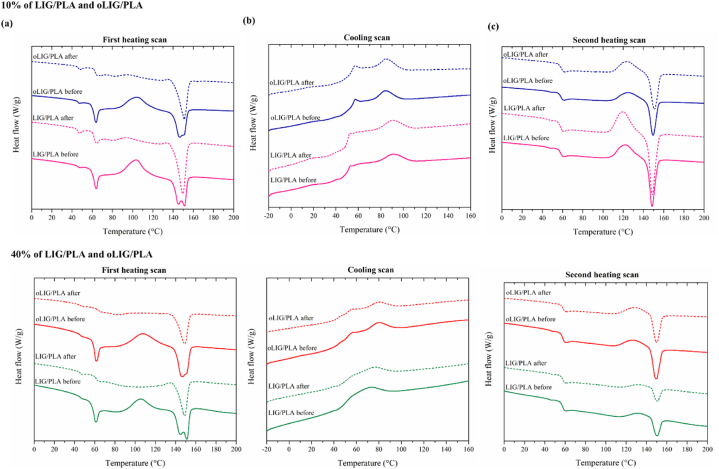
DSC curves of (a) first heating scan, (b) cooling scan, and (c) second heating scan of LIG/PLA and oLIG/PLA composites, 10 % weight (top) and (b) 40 % weight (bottom).

The temperatures obtained during the degradation process of the polymer composites, as indicated by the changes in T_g_ (glass transition temperature), T_cc_ (cold crystallization temperature), T_m1_ (melting temperature), and T_m2_ (second melting temperature), provide valuable insight into the effect of fungal treatment on the thermal properties of the composites. DSC analysis was performed to investigate the thermal characteristics of lignin-PLA (LIG/PLA) and oxypropylated lignin-PLA (oLIG/PLA) (10 % and 40 %) before and after the degradation process. All DSC curves are shown in Fig. 4. In the first heating thermogram (Fig. 4a), after the T_g_ curve, the first peak (in the range between 95 °C and 115 °C), belongs to the cold crystallization peak, followed by the second endothermic peak, which indicates the melting-recrystallization-melting peak of PLA. The two melting peaks observed during the LIG/PLA and oLIG/PLA endothermic process can be attributed to different levels of PLA crystal perfection. The first melting peak corresponds to the less perfect PLA crystal, while the second peak signifies more perfect PLA crystals. These observations align with the findings reported by Mu et al. [54].

The temperature values associated with the evaluation of the T_g_, T_cc_, and T_m_ peaks during the first and second heating scans are provided in Table 1. Interestingly, following the degradation process, all examined composites exhibited significant changes in temperature values. Notably, upon treatment with fungi, there was a substantial reduction in the cold crystallization temperature (T_cc_) for both 10 % LIG/PLA and 40 % oLIG/PLA composites, with T_cc_ values shifting from 103.4 °C to 94.0 °C and from 103.9 °C to 95.0 °C, respectively (Table 1). It is worth noting that for composites with 40 % LIG and oLIG in PLA, no T_cc_ peak was observed after the degradation process during the first heating scan. This absence of a T_cc_ peak may be attributed to the significant disruption of the PLA molecular chain by the *laccase* enzyme. Subsequently, in both cases of LIG/PLA and oLIG/PLA composites, an increase in T_cc_ was observed during the second heating scan after the degradation treatment, indicating that the degradation process can lead to a reduction in the molecular weight of PLA. Lower molecular weight PLA chains tend to crystallize at higher temperatures. These findings align with previous studies conducted by Pyda et al. and Camino et al. [55,56].

**Table 1:** DSC parameter values of 10 % and 40 % of LIG/PLA and oLIG/PLA composites: T_g_ – glass transition temperature, T_cc_ – cold crystallization temperature, and T_m1_ and T_m2_ – melting temperatures from the first heating scan. The standard deviation is in the parenthesis.

Sample		First scan				Second scan		
		T_g_	T_cc_	T_m1_	T_m2_	T_g_	T_cc_	T_m_
10 % LIG/PLA	Before	59.6 (0.6)	103.4 (0.8)	145.1 (0.9)	151.6 (0.3)	57.9 (0.4)	117.3 (2.7)	148.6 (0.4)
	After	60.5 (0.3)	94.0 (1.1)	–	149.6 (0.9)	57.6 (1.0)	118.8 (2.8)	149.8 (0.8)
10 % oLIG/PLA	Before	60.1 (0.2)	103.9 (1.8)	146.8 (0.2)	150.9 (0.3)	58.2 (1.3)	120.7 (2.5)	149.5 (0.5)
	After	58.3 (1.1)	95.0 (2.3)	–	151.0 (0.8)	58.4 (0.3)	122.8 (3.3)	150.9 (1.4)
40 % LIG/PLA	Before	57.9 (0.9)	105.2 (4.3)	144.1 (0.4)	151.4 (0.7)	59.4 (0.6)	130.8 (1.9)	150.3 (0.6)
	After	60.1 (1.3)	–	–	149.0 (1.0)	57.7 (0.5)	134.5 (2.4)	150.8 (0.4)
40 % oLIG/PLA	Before	58.4 (0.8)	107.1 (2.0)	146.9 (0.6)	151.0 (0.5)	57.4 (0.9)	117.5 (5.1)	149.5 (0.4)
	After	58.9 (1.2)	–		149.5 (1.1)	57.7 (0.4)	121.4 (4.2)	150.1 (0.7)

In the context of melting transition temperatures, the T_m2_ peak (representing a secondary melting transition) disappeared after treatment, suggesting potential alterations in the crystalline structure and melting behavior. The calculation of T_g_ (glass transition temperature) from the second heating scan revealed no significant difference before and after the degradation process. These temperature variations during the degradation process indicate potential interactions between the fungi and the polymer composites. The observed changes in T_cc_ and T_m_ imply modifications in the molecular mobility and chain dynamics of the composites, possibly arising from the degradation and reorganization of the polymer matrix.

### 3.3 Microscopic analysis of surface roughness

The microscopic analyses were performed using a KEYENCE VHX-6000 after the degradation process, and the selected micrographs are presented in Fig. S2. The surface roughness increased with higher lignin content in the PLA matrix. In the case of the 40 % weight percentage, the rate of composite degradation was relatively higher than the lower weight percentage. Comparing LIG and oLIG composites, the LIG/PLA surface exhibited more significant changes compared to oLIG/PLA composites. The fungi tend to decompose the unmodified lignin, as the oxypropylation reaction changed the chemical environment in the lignin, inhibiting the fungal growth towards the degradation process.

**Fig. 5:**
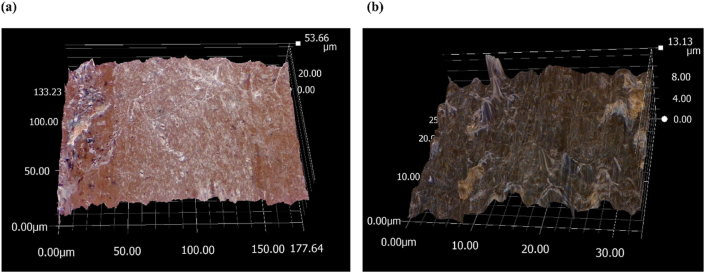
Roughness measurement diagram of the surface after degradation treatment of (a) 40 % LIG/PLA and (b) 40 % of oLIG/PLA.

The microscopic surface roughness of 40 % LIG/PLA and 40 % oLIG/PLA strips after the initial eight weeks of colonization with fungi in a hydrated environment is presented in Fig. 5. The micrographs show that the fungi colonization penetrates LIG, oLIG and PLA composites, resulting in surface roughness. The active *laccase* enzyme in *Trametes versicolor* may be capable of degrading the unmodified lignin (LIG) and oxypropylated lignin (oLIG) composites with PLA even within the initial eight weeks of incubation time. Such a degradation process can be seen through the formation of cavities in the polymer composites in Fig. 5. The surface roughness results indicated that the penetration of the fungi is more prominent on the surface of LIG/PLA composites, as the roughness is larger (approximately 53.66 μm) compared to oLIG/PLA (approximately 13.13 μm). The observation suggests that the fungi grew faster on LIG/PLA, resulting in the secretion of a higher quantity of extracellular *laccase* enzymes to degrade the composites through viable reduction and oxidation reaction compared to its modified counterpart (oLIG/PLA).

The examined composites did not show significant weight loss, but the FTIR and DSC results indicated chemical changes during the eight-week degradation process. The higher roughness of LIG/PLA compared to the oxypropylated counterpart was also observed. Studying the enzyme's interaction with composite components could reveal the underlying interactions and amino acids involved in the degradation process. Molecular docking simulations can provide significant insights into experimental findings and propose key interactions at the atomic scale and the docking results are discussed in the section.

### 3.4 Binding analysis of GGE, oGGE, and LA with Lac by molecular docking

The present study employed computational molecular docking simulations to analyze the individual binding interactions between the active enzyme *Lac* from *Trametes versicolor* fungi and ligands such as unmodified lignin model (GGE), oxypropylated lignin model (oGGE), and lactic acid. The goal was to determine the affinity of the *Lac* fungal enzyme to these ligands within their catalytic sites through molecular docking.

Several molecular docking simulations have been conducted to evaluate the activity of various enzymes that potentially degrade the various polymers, including polyethylene, aromatic hydrocarbons, polyesters, and dyes [[23], [24], [25], [26], [27], [28], [29], [30],^32^,^33^]. In these works, *laccase* is the predominant enzyme used to degrade polymers and dyes due to its oxidoreductive mechanisms. In particular, Sánchez et al. compared different active enzyme substrates for their ability to degrade polyethylene [29]. The results revealed that the *Lac* enzyme from *Trametes versicolor* exhibited the strongest binding interaction with polyethylene compared to other enzymes, including *LiP* (lignin peroxidase), *MnP* (manganese peroxidase), and *Cut* (Cutinase), as determined through molecular docking simulations. The *Lac* enzyme is composed of four copper (Cu) ions, with one of these Cu ions playing a major role in the degradation of polyethylene. Sánchez et al. also proposed that the *Lac* enzyme can break down the polyethylene through a series of reactions including oxidation and reduction. Similarly, we assume that the composites such as lignin, oxypropylated lignin, and poly(lactic acid) undergo oxidation and reduction reactions, cleaving the ester and ether bonds which leads to the liberation of carboxylates and alcohols.

**Fig. 6:**
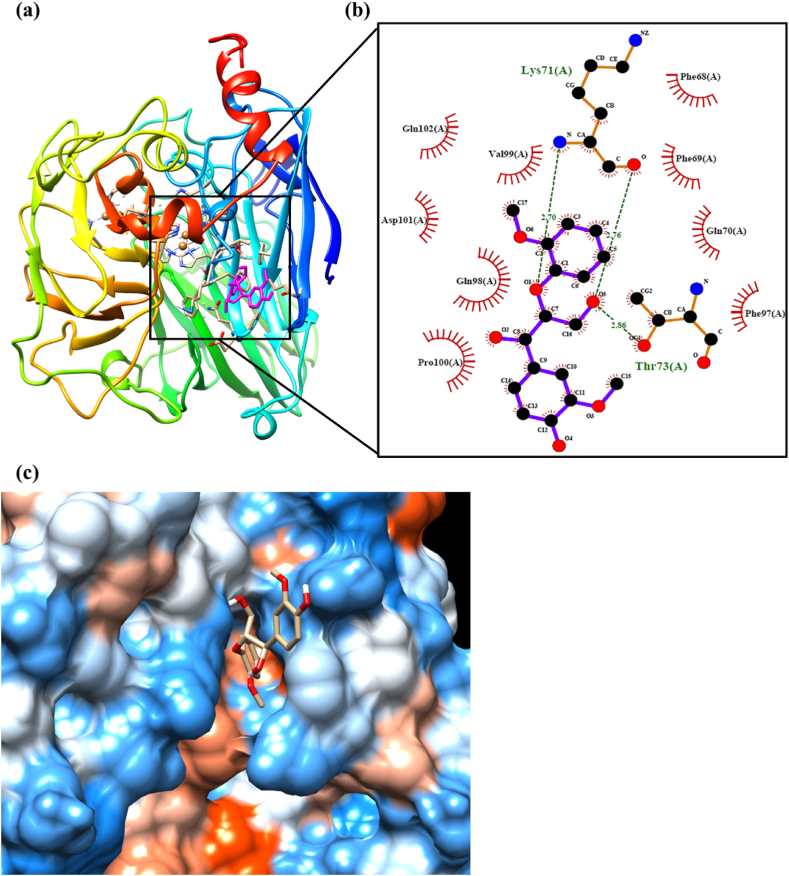
Molecular docking results of the most stable binding interaction of *Lac* with GGE (unmodified), (a) docked Lac-GGE configuration with active site (inside the box) where GGE was illustrated with magenta and *Lac* depicted on the secondary ribbon structure according to n-terminus in blue to c-terminus in red (obtained from UCSF Chimera), (b) illustrated active site with different amino acids from *Lac* through hydrophobic and hydrophilic interactions to the GGE ligand (software LigPlot+), and (c) hydrophobic surface representation of the *Lac* enzyme with GGE ligand binding cavity (from UCSF Chimera).

**Fig. 7:**
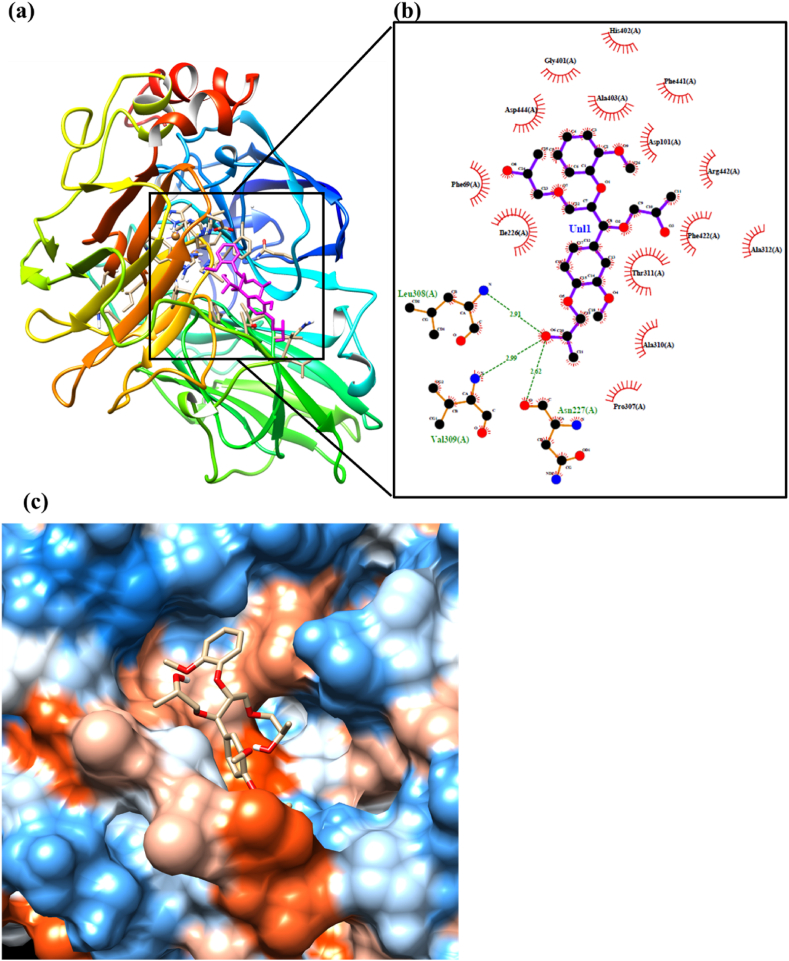
Molecular docking results of the most stable binding interaction of *Lac* with oGGE (oxypropylated), (a) docked *Lac*-oGGE configuration with active site (inside the box) where oGGE was illustrated with magenta and *Lac* depicted on the secondary ribbon structure according to n-terminus in blue to c-terminus in red (obtained from UCSF Chimera), (b) illustrated active site with different amino acids from *Lac* through hydrophobic and hydrophilic interactions to the oGGE ligand (software LigPlot+), and (c) hydrophobic surface representation of the *Lac* enzyme with oGGE ligand binding cavity (from UCSF Chimera).

**Fig. 8:**
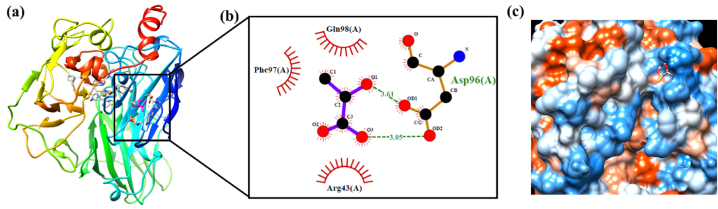
Molecular docking results of the most stable binding interaction of *Lac* with LA (unmodified), (a) docked *Lac*-LA configuration with active site (inside the box) where LA was illustrated with magenta and *Lac* depicted on the secondary ribbon structure according to n-terminus in blue to c-terminus in red (obtained from UCSF Chimera), (b) illustrated active site with different amino acids from Lac through hydrophobic and hydrophilic interactions to the LA ligand (software LigPlot+), and (c) hydrophobic surface representation of the *Lac* enzyme with LA ligand binding cavity (from UCSF Chimera).

Fig. 6, Fig. 7, Fig. 8 illustrate the binding activity of *Lac* with ligands, providing further details on the amino acids that primarily participate in the interaction with ligands during the degradation process. The binding energy of the enzyme-ligand complexes was calculated to determine the optimal enzyme-ligand interaction, which could be correlated to the degradation of composites. The calculated binding energy for each system is presented in Table 2, and the values indicated that the unmodified GGE lignin model had higher binding energy than other ligands, such as oGGE and LA. The energy order was −5.96 > −5.76 > −4.91 kcal/mol, respectively. Interestingly, LA was more strongly bound to the *Lac* active site than *Lac*-oGGE, which showed the least binding interaction of oGGE with *Lac*. These findings emphasize that the oxypropylation modification of lignin significantly reduces the degradation of composites while demonstrating good thermal and mechanical properties and compatibility with PLA, as previously indicated in our work [34]. The order of binding energy obtained from molecular docking simulations is in agreement with the experimental findings described in this study.

**Table 2:** The estimated binding energy (Kcal/mol) and binding affinity (μM) of each ligand investigated in this study using the molecular docking method.

Ligand	Binding energy (Kcal/mol)	Binding affinity (μM)
Unmodified lignin (GGE)	−5.96	42.62
Oxypropylated lignin (oGGE)	−4.91	250.57
Lactic acid (LA)	−5.76	59.77

The strength of interaction between enzyme and ligand is expressed by the binding strength or inhibition constant [29]. The binding affinity values obtained for each examined system are presented in Table 2. Generally, lower binding affinity values indicate stronger attraction between the enzyme and ligand [57], whereas higher values indicate weaker binding. Table 2 shows that the oGGE ligand had a higher binding affinity value with the *Lac* enzyme, showing that oxypropylated lignin exhibited the least interaction with the enzyme. Moreover, the corresponding binding affinity value (250.57 μM) is over four times higher than the *Lac*-LA interaction and almost six times higher than the *Lac*-GGE interaction. The final binding affinity correlation scores of the examined systems are 42.62 > 59.77 > 250.57 μM, for Lac-GGE > Lac-LA > Lac-oGGE, respectively. The obtained order associated with the binding affinity is consistent with the binding energy values.

### 3.5 Lac enzyme interaction with unmodified lignin model compound (GGE)

Table 3 presents the amino acid residues that strongly bind with the GGE ligand, and their corresponding interactions are depicted in Fig. 6b. Two amino acids, Lysine (LYS) and Threonine (THR), form three predominant hydrogen bond interactions, with LYS forming shorter hydrogen bonds with the GGE ligand. The hydrogen bonds between the amino acids and the GGE ligand (Fig. 6b) suggest that the amine (NH_2_) and O (acid) groups of LYS contributed to bonding with O_β_ in GGE and the acid group in LYS interacting with the γ-OH group. The amine group in LYS acts as a donor, while the acid group in LYS serves as an acceptor for hydrogen bonds. Similarly, the THR amino acid forms a hydrogen bond with the γ-OH group in GGE, with a distance of 2.86 Å, acting as a proton donor.

**Table 3:** The list of selected amino acids strongly bound to GGE ligand through hydrogen bonds and hydrophobic interaction and the distance between amino acids and GGE ligand is depicted with Angstrom, and the hydrogen bond donor angle is mentioned with the degree.

Hydrogen bonds	Amino Acids	Distance (Å)	Donor Angle (°)
1	LYS	2.70	140.30
2	LYS	2.76	159.42
3	THR	2.86	121.55
**Hydrophobic interaction**			
1	PRO	3.00	
2	PHE	3.27	
3	PHE	3.27	
4	VAL	3.34	
5	GLN	3.64	
6	VAL	3.97	

Furthermore, hydrophobic interactions occur via several amino acids, and Table 3 highlights the selected interactions. Among these amino acids, Proline (PRO) tends to form a shorter hydrophobic interaction (3.00 Å) than other amino acids, and Valine (VAL) exhibits the least interaction, with the highest bond distance of 3.97 Å. Other amino acids, such as Phenylalanine (PHE), Valine (VAL), and Glutamine (GLN), also contribute to the hydrophobic interactions with GGE.

### 3.6 Lac enzyme interaction with oxypropylated lignin model compound (oGGE)

The hydrogen bonds and hydrophobic interactions between *Lac* and oGGE were evaluated (shown in Fig. 7) to understand the impact of the oxypropylation reaction on the binding interaction. Table 4 shows the amino acids that participated in these interactions. Among these amino acids, ASN, LEU, and VAL strongly interacted with oGGE, and ASN formed a shorter hydrogen bond than LEU and VAL. Notably, these amino acids are bound to only the hydroxyl group in the oxypropylated chain. Of these amino acids, VAL and LEU contributed as proton donors through their amine group, whereas ASN acted as a proton acceptor through the acid group. Furthermore, several amino acids participated in hydrophobic interactions with the oGGE ligand, and Table 4 presents the selected amino acids involved in these interactions. ASN had a stronger hydrophobic interaction with oGGE compared to other amino acids, and the order of hydrophobic interaction of different amino acids, in terms of distances, was ASN > ASP > PHE > THR > ILE.

**Table 4:** The list of selected amino acids strongly bound to the oGGE ligand through hydrogen bonds and hydrophobic interaction and the distance between amino acids and the oGGE ligand is depicted with Angstrom, and the hydrogen bond donor angle is mentioned with the degree.

Hydrogen bonds	Amino Acids	Distance (Å)	Donor Angle (°)
1	ASN	2.62	142.17
2	LEU	2.91	113.88
3	VAL	2.99	134.37
**Hydrophobic interaction**			
1	ASN	3.24	
2	ASP	3.33	
3	PHE	3.33	
4	THR	3.44	
5	ILE	3.62	
6	ILE	3.82	

### 3.7 Lac enzyme interaction with lactic acid (LA)

Although LA also interacts with *Lac* enzyme like other ligands (GGE and oGGE), the number of interactions is relatively low due to the small size of the PLA model used in the study. The results of the hydrogen bonding and hydrophobic interactions between LA and PLA are presented in Table 5 and illustrated in Fig. 8. The ASP (Aspartic acid) amino acid is predicted to form a strong bond with the LA ligand, with distances of 3.05 Å and 3.61 Å. The hydroxyl proton and acid proton in LA act as donors to form hydrogen bonds with ASP amino acid. In addition, ARG (Arginine) and GLN (Glutamine) were found to interact with LA via hydrophobic interactions, with distances of 3.12 Å and 3.35 Å, respectively.

**Table 5:** The list of selected amino acids strongly bound to the LA ligand through hydrogen bonds and hydrophobic interaction and the distance between amino acids and LA ligand is depicted with Angstrom, and the hydrogen bond donor angle is mentioned with the degree.

Hydrogen bonds	Amino Acids	Distance (Å)	Donor Angle (°)
1	ASP	3.05	145.03
2	ASP	3.61	172.36
**Hydrophobic interaction**			
1	ARG	3.12	
2	GLN	3.35	

Overall, the molecular docking results revealed that the modification of lignin, particularly oxypropylation, significantly reduced the binding ability with the *Lac* enzyme, indicating poor degradation of composites with PLA as the *Lac* enzyme primarily degrades the phenolic groups in lignin. It should be noted that the model compounds used for lignin and lactic acid are relatively small compared to the corresponding polymers used in composite preparations. Nevertheless, this molecular docking study provides valuable insight into the underlying atomic-level interactions that support the understanding of degradation mechanisms in materials.

## 4 Conclusions

This study focused on investigating the initial degradation of unmodified lignin (LIG) and oxypropylated lignin (oLIG) incorporated at 10 % and 40 % levels within polylactic acid (PLA) composites under the influence of *Trametes versicolor* fungi in a controlled fungal environment. The degradation process was limited to just eight weeks to capture the incipient stages of decomposition. We observed no significant difference in weight loss during this initial eight-week period. However, in-depth analyses using FTIR and DSC revealed substantial alterations in the chemical and thermal properties of the composites.

The carbonyl index values calculated from FTIR spectra indicated oxidative changes in the C=O groups, resulting in reduced index values after treatment for all cases, including LIG/PLA and oLIG/PLA. Interestingly, while the hydroxyl index increased for unmodified systems (10 % and 40 % LIG/PLA), it decreased for oLIG/PLA, underscoring the impact of oxypropylation on composite degradation. The DSC results provided valuable insights, as the absence of the cold crystallization peak in the first heating scan indicated disruption of the PLA crystallization region by fungal enzymes. However, during the second heating scan, the cold crystallization temperature increased after treatment, possibly due to the formation of low molecular weight PLA chains. Microscopic analysis revealed higher roughness in LIG/PLA compared to oLIG/PLA.

Furthermore, molecular docking simulations using the guaiacylglycerol-β-guaiacyl ether (GGE) model compound for lignin and lactic acid for PLA, with the active *Lac* enzyme from *Trametes versicolor*, provided insights into the binding mechanisms. The binding affinity results showed the following trend: GGE > lactic acid (LA) > oxypropylated GGE (oGGE). The consistency between experimental and molecular docking findings offers a comprehensive understanding of the initial fungal enzyme degradation process involving *Trametes versicolor* in LIG/PLA-based composites. This research contributes to the development of controlled degradation processes for biobased polymer composites with active *laccase*-based microorganisms, supporting sustainable waste management strategies.

## Funding

The authors gratefully acknowledge the 10.13039/501100000780European Commission for funding the InnoRenew project [H2020 WIDESPREAD-2-Teaming grant number 739574] and the Republic of Slovenia [investment funding from the Republic of Slovenia and the European Regional Development Fund]. ESE acknowledges Horizon2020, the 10.13039/501100007601EU Framework Programme for Research and Innovation [Marie Sklodowska-Curie Actions grant number 101031402].

## Data availability statement

Data will be made available on request.

## CRediT authorship contribution statement

**Esakkiammal Sudha Esakkimuthu:** Writing – original draft, Visualization, Validation, Project administration, Methodology, Investigation, Funding acquisition, Formal analysis, Data curation, Conceptualization. **Veerapandian Ponnuchamy:** Writing – review & editing, Writing – original draft, Visualization, Validation, Software, Methodology, Investigation, Formal analysis, Data curation, Conceptualization. **Marica Mikuljan:** Writing – review & editing, Visualization, Validation, Methodology, Investigation, Data curation. **Matthew Schwarzkopf:** Writing – review & editing, Supervision. **David DeVallance:** Writing – review & editing, Supervision.

## Declaration of competing interest

The authors declare that they have no known competing financial interests or personal relationships that could have appeared to influence the work reported in this paper.
