# Supplementary material for: Fungal enzyme degradation of lignin-PLA composites: Insights from experiments and molecular docking simulations
Source: Heliyon. 2023 Dec 17;10(1):e23838. doi: 10.1016/j.heliyon.2023.e23838 (PMC10772188; doi:10.1016/j.heliyon.2023.e23838)
Supplement: Multimedia component 1 [file mmc1.docx]

**Fungal Enzyme Degradation of Lignin-PLA Composites: Insights from experiments and molecular docking simulations**

Esakkiammal Sudha Esakkimuthu, Veerapandian Ponnuchamy, Marica Mikuljan, Matthew Schwarzkopf, David DeVallance

**
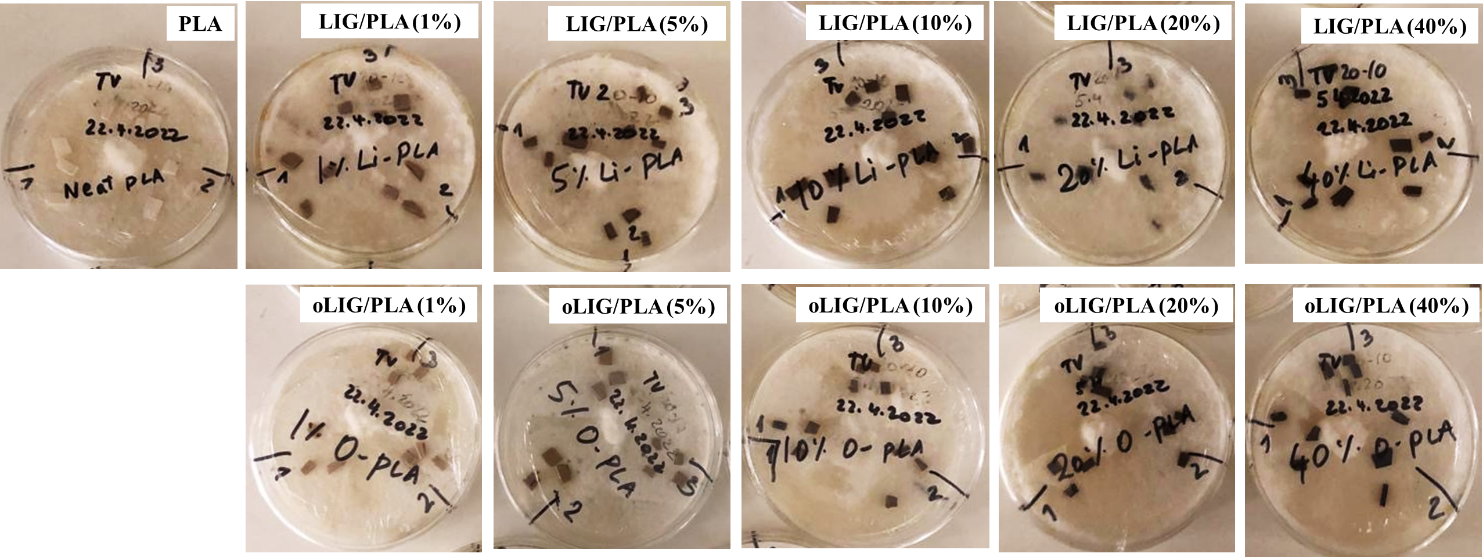
**

**Fig. S1.** The composite samples (neat PLA, LIG/PLA, and oLI/PLA) inoculated in the *Trametes versicolor* fungal media at different weight % of lignin (1%, 5%, 10%, 20%, and 40%)


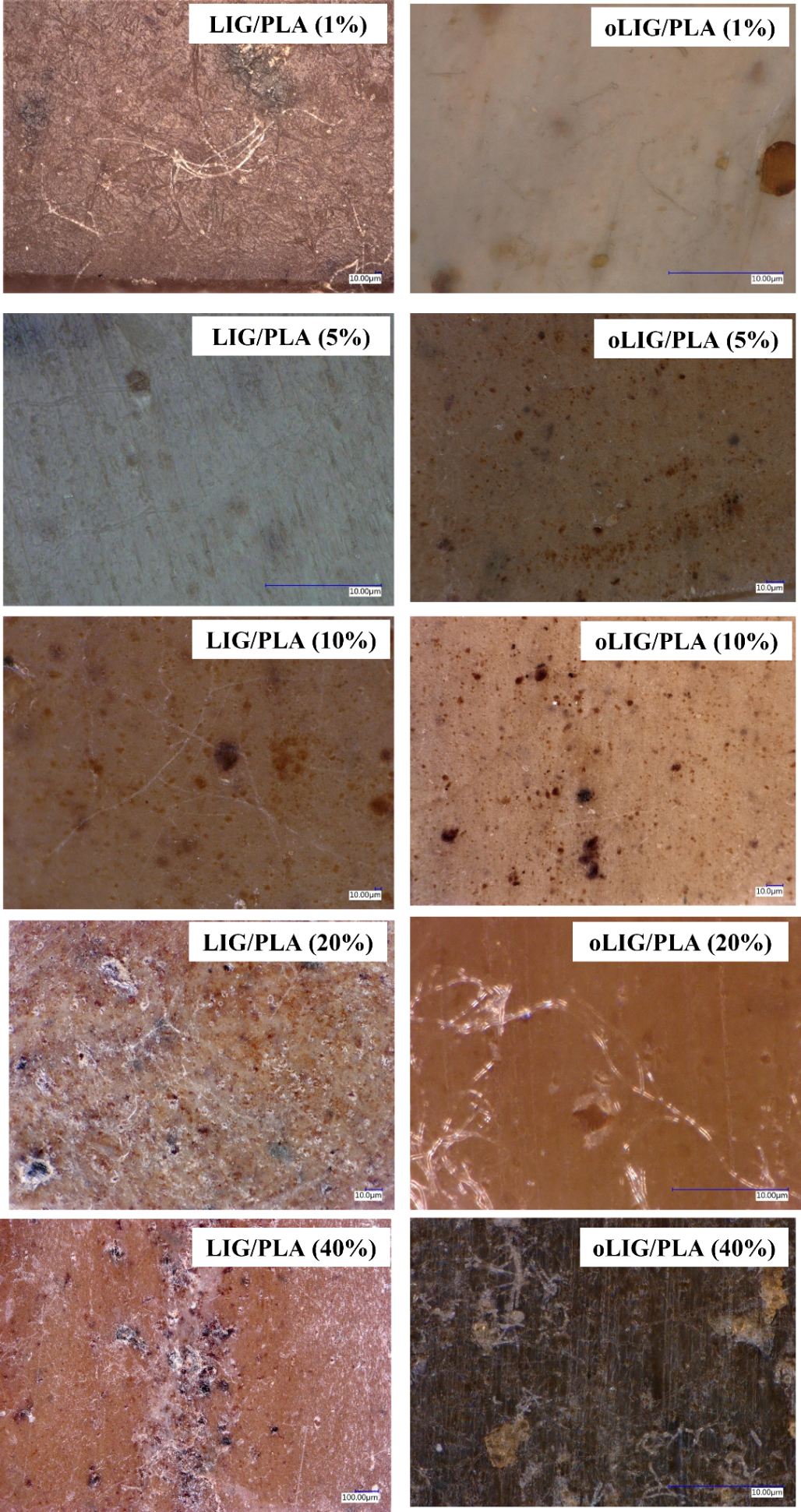


**Fig. S2**. Microscopic surface morphology of the lignin and oxypropylated lignin with PLA composites at different weight percentages after the degradation process.
